# Reaching out to stakeholders: The use of knowledge terminology on the websites of Australian public hospitals

**DOI:** 10.1186/s12913-020-05798-y

**Published:** 2020-10-26

**Authors:** Andrej Miklosik, Nina Evans

**Affiliations:** 1 Marketing Department, Faculty of Management, Comenius University in Bratislava, Bratislava, Slovakia; 2 UniSA STEM, University of South Australia, Adelaide, Australia

**Keywords:** Communication, Hospitals, Knowledge, Knowledge culture, Knowledge terminology, Websites

## Abstract

**Background:**

The objective of the study described in this article was to examine whether, and to what extent, Australian public hospitals use knowledge terminology, i.e. a body of knowledge-related terms, on their websites. The paper also discusses the difference in the level of such communication between large and small hospitals, the factors affecting the use of the knowledge-related terms in the communication and the similarities/differences between the use of knowledge terms in Australian public hospitals and large/small companies in Australia.

**Methods:**

151 Australian public hospitals were included in the research sample: 51 large and 100 small hospitals. Using the method of content analysis, websites mentioning knowledge creation, knowledge sharing, knowledge implementation, and knowledge retention were identified, along with the number of these mentions. Descriptive statistics and chi square test of independence were used to provide answers to four research questions.

**Results:**

Of the 151 hospitals included in the sample, 30 had no website and 62 (50 small and 12 large) had a single page website. The study found that there are differences between Australian public hospitals regarding the level of their knowledge communication on their websites, both between small and large hospitals and between the individual hospitals within the large and small hospital groups.

**Conclusions:**

A well-known saying goes “For the mouth speaks what the heart is full of”. Effective communication of knowledge-related terminologies to both internal and external stakeholders, i.e. the parties who access the websites, is therefore an indication of a knowledge focus in the public hospitals. Large hospitals are generally more active in communicating knowledge terms, although there are some exceptions. Some of the small hospitals can lead by example, but most of them do not include knowledge terminology in their communication on websites.

## Background

The health industry is facing increasing pressure to reduce costs and increase quality of healthcare. This pressure is forcing dramatic changes throughout the industry. Literature posits that a ‘paradigm shift’ is occurring due to the spiralling costs, financial constraints, increased emphasis on accountability and transparency, changes in education, growing complexities of biomedical research, new partnerships in healthcare, and advances in information technologies (IT) [1]. Links between the application of advanced IT systems, supported knowledge and skills, and the quality of customer care have been established [2]. In today’s healthcare environment there is an emphasis on prevention and managing a patient’s health and wellbeing throughout their life [1].

The medical sector makes up a large proportion of a country’s budget and gross domestic product (GDP). Therefore, any improvements to this sector will lead to benefits for a country [3]. Healthcare in Australia comprises multiple agencies, including public and private hospitals, outpatient services, general practice, and allied health. Traditionally minimal exchange of knowledge occurs between healthcare providers from different sectors, partly due to the complexity of information security and privacy [4]. However, the paradigm shift in the healthcare industry necessitates a focus on interaction, collaboration, and increased sharing of information and knowledge [1]. Knowledge is the most important asset for healthcare organisations to create sustainable competitive advantage and ensure success in the changing environment [5]. The way knowledge is managed should therefore receive much attention from the health industry [6].

At the same time, organisations are challenged to increase the transparency of their policies and communicate their commitment, efforts, and results to different groups of stakeholders via online channels [7, 8]. These stakeholders include patients, their families, suppliers, doctors, nurses and hospital administrators. It has been confirmed that sharing project-related knowledge with various stakeholders encourages their engagement and collaboration [9]. Improving the access to relevant information on how the organisation manages its knowledge positively impacts the sustainability of strategic relationships with them [10].

The main objective of this study was to investigate whether—and to what extent—Australian public hospitals use terms related to knowledge, thereby reflecting a knowledge focus. This is of great interest because of the expectation that a knowledge focus is reflected not only in internal processes, but also in the way organisations communicate online with their target audiences. The following research questions have been defined:

- 1. Do Australian public hospitals include knowledge terminology on their websites?
- 2. Is there any difference in the extent to which knowledge-related terms are used on the websites between large and small hospitals and within the groups of large and small hospitals?
- 3. What other factors affect the use of knowledge-related terms in the communication?
- 4. Is the use of knowledge terms on the websites of Australian public hospitals similar or different to that in large companies and SMEs in Australia?

### Importance of knowledge in hospitals

Hospitals are regarded as ‘knowledge-intensive (KI) organisations’, where work is mainly of an intellectual nature, where knowledge is very important and most employees are knowledge workers [5]. Both explicit (formalised, written) and tacit (knowledge in people’s heads) forms of knowledge are present in hospitals [11]. Explicit knowledge is available in medical journals, research reports, and industry publications. Tacit knowledge is found in the minds of highly specialised practitioners, such as neurosurgeons or cardiac arrest specialists [3]. Healthcare is experiencing an exponential growth in the scientific understanding of diseases and their treatment. To deliver high-quality healthcare doctors need to access, interpret, and share the localised medical knowledge [12], which requires significant investment in these knowledge assets [1]. However, according to [13] the growth of knowledge is not congruent with the ability to effectively disseminate, translate, and apply current healthcare knowledge in clinical practice.

Hospitals use knowledge as a critical factor in running the administrative, financial, and clinical aspects of their management [13]. Knowledge assets in hospitals therefore include knowledge regarding patient care, medical needs, the operating environment, and technologies that can be utilised in routine medical and healthcare management [1]. Knowledge is one of the most important assets in hospitals, guiding clinical care and quality improvement, along with directing population health management [14–16]. Medical knowledge is important for clinical decision-making, teaching, continuing professional development, research, and delivering better outcomes for patients [17]. This knowledge is sourced from discussions with other clinicians, medical records, test results, and conversations with patients and families [18, 19]. Clinicians must navigate a large quantity of information and knowledge to provide the best evidence-based care available [18, 19]. As a result, the importance of medical knowledge has been highlighted in the ongoing initiatives to promote evidence-based medicine and clinical quality improvement [12].

Knowledge is managed in hospitals through the following process:

- i) Knowledge creation and elicitation:
  Knowledge creation refers to the ability to develop new ideas or solutions [20], which can be done through discovery at individual and group level. Once such knowledge is generated, it can be made available to external sources.
- ii) Knowledge capture and storage:
  Once gathered, knowledge needs to be captured and stored to allow for dissemination and transfer. The knowledge can be *codified, stored and reused*, or it can be *personalised* through dialogue between individuals.
- iii) Knowledge transfer and dissemination:
  Once knowledge is stored, it has to be made available for access by the various stakeholders. Effective *knowledge sharing* occurs when a person is able and willing to assist others, and also to learn from other individuals [20].
- iv) Application and exploitation of knowledge resources:
  Knowledge assets provide value if they are used for clinical decision-making. Knowledge *application* refers to predefined routines, e.g. the basic diagnosis when a patient enters the hospital includes measuring blood pressure, pulse rate, etc. Knowledge *exploitation* calls for the use of knowledge resources on an ad-hoc basis in random decision-making scenarios [3].

Top management involvement and support for knowledge initiatives is vital in hospitals [3]. Transformational leaders may become the source of inspiration and serve as role models for organisational members to share their valuable knowledge with others [21].

### Knowledge-focused organisations

There is an old saying: ‘For the mouth speaks what the heart is full of’. What the ‘organisation mouth’ speaks of, is an indication of its focus. An organisation can create a favourable environment for successful management and exploitation of its knowledge. Such a ‘knowledge focus’ relates to culture, values, and organisational structure. The organisational structure must support the organisation to deliver the right information to the right people at the right time [22]. Social norms such as openness and teamwork—where cooperation is fundamental—are key characteristics of knowledge intensive organisations [5]. De Long [23] identified four ways in which organisational culture influence the way they deal with organisational knowledge. The culture (i) shapes assumptions about what constitutes knowledge and what knowledge is worth managing; (ii) mediates the relationships between levels of knowledge; (iii) creates the context for social interaction; (iv) shapes the processes by which new knowledge is created, validated, and disseminated in organisations. Prevailing organisational values and beliefs may promote or hinder an organisation’s capacity for creating, sharing, and implementing knowledge [20].

It is essential and challenging for hospitals to be able to create and demonstrate an awareness of the importance of knowledge creation, acquisition, collection, dissemination, sharing, implementation, and exploitation. Hospitals especially need to develop a knowledge focus that enables and encourages the process of shifting knowledge among people [20]. The biggest challenge for most KM efforts lies in developing a culture of collaboration, trust, innovation, problem solving, and openness [24]. This will be evident in the terminology—i.e. the ‘organisational words’—spoken in communication with stakeholders of the hospital [5], as discussed in the next section.

### Communicating knowledge terminology via the hospital website

A hospital website represents one of the main channels for communication between the organisation and its stakeholders [25]. The website can be perceived as an information system that connects people to experts rather than providing knowledge directly [26]. Cannoy [27] conducted an investigation of websites of 10 hospitals listed in U.S. News and World Report’s Best Hospitals of 2004 Honour Roll, as well as a random selection of seven other hospital sites to determine the features that enhance communication between the partners in healthcare. They suggest that the internet is widely utilised by a critical mass to enhance communication and that internet technologies can be a strategic asset for hospitals, as they can impact overall enterprise performance efforts through communication-enhancing features with the potential to increase communication between the hospital and its partners.

Dal Buono [28] observe that the often ineffective and inconsistent online presence, when managed well, indicates that an institution is in step with the times and that it has a close relationship with its stakeholders and members. The way in which a hospital’s knowledge focus is communicated through its publicly available website will provide an indication whether knowledge is valued and managed in the organisation. In hospitals where managers and leaders are committed to providing support for knowledge-enhancing activities, and where they ‘walk the talk’ by managing their own knowledge, the message will be effectively communicated to existing and prospective employees, as well as clients. All parts of these hospitals will support knowledge-related activities and the knowledge-focused culture will therefore be visible in the regular inclusion of terms such as knowledge (identification), creation (learning), sharing (collaboration), retention, and implementation-related terms on a hospital’s website [29].

### The impact of organisational size on the knowledge focus

In today’s highly competitive market environment, organisations of all sizes have to manage knowledge effectively and efficiently to survive. Knowledge-related issues occur across the board, but different sized organisations address these issues differently [30]. Xu [31] found that larger organisations manage its knowledge more consciously and systematically than smaller businesses and prefer knowledge transfer via technology. Miklosik [29] agree that large organisations use more advanced IT technologies to manage their knowledge. According to [32] the critical success factors to manage knowledge in small and medium organisations are management leadership and support; culture; IT; strategy and purpose; measurement; organisation infrastructure; processes and activities; motivational aids; resources; training and education; and human resource management. Xu [31] determined that smaller sized organisations prefer a personal approach. Miklosik [29] add that they are sometimes also focused on information application, but the correct information is often not available. This causes duplication and re-invention of information that may be readily available elsewhere.

## Methods

Content analysis is defined as a “research technique for making replicable and valid inferences from texts (or other meaningful matter) to the contexts of their use” [33], i.e. systematically evaluating texts (e.g. documents of various forms and verbal communication) looking for specific words and phrases, followed by coding and categorizing to induce assumptions. Content analysis was therefore used to record the number of times specific knowledge terms are referenced in media, specifically to collect and analyse data from the websites of Australian public hospitals.

### Selecting the research sample

Purposive sampling was used to select the entities for the research, using the “Hospital resources 2016–17: Australian hospital statistics” list [34]. The list contains 695 public Australian hospitals from all six Australian states and two territories. 151 hospitals and their websites were included in the research sample, consisting of 100 small hospitals and 51 large hospitals from all states and territories. The size of the hospitals referred to the number of beds, with the large hospitals having more than 6 beds and small hospitals having 6 or fewer beds. Other criteria included the location (state), characteristics of the website, e.g. its complexity and the existence of an own domain. Both public acute hospitals and public psychiatric hospitals were included in the research sample. Private hospitals were excluded from the study. The number of hospitals included from different states and territories were as follows: Australian Capital Territory (ACT) (1); New South Wales (NSW) (33); Northern Territory (NT) (1); Queensland (QLD) (22); South Australia (SA) (12); Tasmania (TAS) (11); Victoria (VIC) (33) and Western Australia (WA) (38).

### Variable selection

Ten variables were recorded:

- 1) Size – the number of beds.
- 2) State – ACT; NSW; NT; QLD; SA; TAS; VIC and WA.
- 3) P or O – ‘P’ indicates that the website is a complex portal consisting of multiple pages; ‘O’ represents a one-page website that does not have a deeper structure and shows all the information about the hospital on one page.
- 4) Dom – the existence of an own domain was identified. ‘2^nd^’ indicated that the hospital website URL looks e.g. like ‘shvs.org.au’ and thus, runs on its own 2^nd^ level domain; ‘3^rd^’ was used if the website is operated on an own subdomain name before the main domain name, such as fionastanley.health.wa.gov.au; ‘no’ was used if the hospital website resides on a general domain used by multiple entities, such as the health network domain with the homepage URL being e.g. ‘health.qld.gov.au/townsville’.
- 5) K – the total number of documents containing the word ‘knowledge’.
- 6) KC – the total number of documents containing terms related to knowledge creation.
- 7) KR – the total number of documents containing terms related to knowledge retention.
- 8) KS – the total number of documents containing terms related to knowledge sharing.
- 9) KM – the total number of documents containing terms related to knowledge management.
- 10) Total – represents the sum of values for variables 6 to 9, i.e. the total number of documents containing any of these knowledge terms.

### Data collection

To collect the data for variables 3 (P/O) and 4 (Dom), observation and visual analysis were used. Links on the website were followed to see whether there is a deeper structure behind the first page (homepage). Sitemap was opened if available to confirm the findings.

To gather values for variable 5, a series of searches using the Australian version of Google was performed. Google.com.au website was used in an incognito mode to enter the search phrases. The keyword ‘knowledge’ was used as a search phrase and the website domain was added to limit the search results to include the examined hospital website only. The search string looked like:

knowledge site:https://fionastanley.health.wa.gov.au

The number of online resources containing this keyword was recorded. The value was further refined by manually checking every page with search results, as Google occasionally includes a page that does not contain the keyword in the search results.

For variables 6 to 9, the same principle was used, however, multiple keyword combinations were entered. For each of the variables, a series of keywords closely related to the examined area was defined and used as search strings. For example, when looking for the number of websites mentioning terms related to knowledge creation (KC), terms such as ‘create knowledge’, ‘generate knowledge’, ‘gain knowledge’ etc. were also included. The search string used looked like:

“generate knowledge” site:https://fionastanley.health.wa.gov.au

The results of this search string would include only pages that contained this exact phrase – both words next to each other. To increase the relevance of results, variations with another word being included in between them were also considered. If a text on a web page reads e.g. ‘gain new knowledge’, this should be considered as communicating about the knowledge creation area. The number of these occurrences were added to the previous value. The modified search string was:

“generate * knowledge” site:https://fionastanley.health.wa.gov.au

The principles introduced in the methodology that was used in the study by [29] were applied to enable a comparison of the results with different industries. The study evaluated three options of collecting the data to analyse the online communications about knowledge terminology. The method of using Google search to determine the number of websites with the terms mentioned was used in both studies, however, in the presented study it was enhanced by including keywords that were separated by another word and expanding the number of keywords for each of the areas. The already referenced [29] study examined three areas based on the three knowledge processes defined [20] —knowledge creation, knowledge sharing, and knowledge implementation. Further adjustments were made to the methodology in the presented study, as one extra area was added to include the topic of knowledge retention. More keywords were also used for each knowledge area. The final keyword list for each of the variable (area) was finalised after extensive testing, where multiple keyword combinations were used to examine the results for the hospitals with the most keyword mentions on their websites. This iterative approach enabled the researches to include the most relevant keywords and not focus on keyword combinations that would not have enough coverage. The keywords list thus reflects the existence of a knowledge focus in an organisation. The researchers are aware that the list is not, and will never be, complete.

We considered the number of websites containing each of the following terms:

#### KC (knowledge creation)

build knowledge, building knowledge, create knowledge, creating knowledge, generate knowledge, generating knowledge, acquire knowledge, acquiring knowledge, improve knowledge, improving knowledge, increase knowledge, increasing knowledge, develop knowledge, developing knowledge, expand knowledge, expanding knowledge, gain knowledge, gaining knowledge, knowledge building, knowledge creation, knowledge expansion, knowledge acquisition, knowledge generation

#### KR (knowledge retention)

knowledge retention, knowledge capture, knowledge base, retain knowledge, retaining knowledge, retention of knowledge, capture knowledge, capturing knowledge, body of knowledge

#### KS (knowledge sharing)

knowledge sharing, knowledge transfer, knowledge exchange, knowledge dissemination, knowledge application, sharing knowledge, apply knowledge, applying knowledge, share knowledge, dissemination of knowledge, disseminate knowledge, transfer knowledge, transfer of knowledge

#### KM (knowledge implementation/management)

Implement knowledge, manage knowledge, managing knowledge, knowledge implementation, knowledge management

### Data preparation

Before the analyses were conducted, the data were cleaned and prepared. Descriptive statistics was applied to determine the total keyword occurrences and illustrate the difference in the use of knowledge-related terminology on the websites between different hospitals.

To allow for significance testing, numerical variables were recoded as categorical variables as follows:

- **Size:** this was recoded with categories *Large* (more than 6 beds) or *Small* (up to 6 beds). The decision to use 6 beds as the cut-off came from the data which showed two clear groupings.
- **MentionedK:** this was recoded as a binary variable indicated either the phrase K was mentioned (counts of at least 1) or the phrase was not mentioned (counts of 0). In the raw data counts of mentions of “K” were dominated by 0 s which severely impacts and limits the statistical analysis possible, hence the choice to convert the variable to binary.
- **MentionedKC, KR, KS, KM, Total:** “Mentioned” binary variables were also created for the remaining phrase counts for KC, KR, KS, KM and Total using the same rationale.

### Testing

The chi-square test of independence was used to analyse the two-way contingency table for each variable combination to determine whether there is an association between two variables of interest. If the result was significant, indicating an association between the categories of the two variables, then post-hoc analysis was applied to identify the categories producing a significant result. In the two-way tables where cell counts equalled 0, violating a fundamental assumption of the chi-square test of independence, Fisher’s Exact Test was used to validate the results of the Chi-Square test.

## Results

### Website communications of Australian public hospitals

Not all small hospitals have a website. In 30 cases (30.0%), their contact information can only be found on a website MyHospitals.com.au operated by the Australian Institute of Health and Welfare that is aggregating contact data of all hospitals in Australia. Of the 70 small hospitals that have their own website, 50 (71.4%) operate a one-page website that contains the address, contact telephone number, and basic information about staff and services offered. Typically, it is not even a standalone website but a page that forms part of a larger website (portal) operated under the domain the Department of Health website of the respective state government or the local council. All the large hospitals have a website, however, 12 out of 51 (23.5%) only have a one-page style website containing no details, articles, media or knowledge.

### Knowledge-related terms used in the website resources

Variable No. 5 (K) can be used to get the first and quick impression of the level of communications using knowledge-related terms on the websites of the examined hospitals. Although the number of references to ‘knowledge’ in communication with the online audience does not uncover the context, it can be used as a good indicator of a knowledge focus.

Most of the small hospitals (85outof100) did not mention the keyword ‘knowledge’ on their websites. However, despite their small size some of these hospitals not only operated their own full-featured website but also communicated with their target audience using knowledge related terms

Descriptive statistics can be used to gain insight into the differences between small and large hospitals. Data comparing the size of entities in each group and the number of times knowledge-related terms are communicated, are displayed in Table 1.

**Table 1:** Initial comparison of both groups using descriptive statistics

Indicator/Value	Beds		Keyword Mentions	
	Small	Large	Small	Large
Maximum	6.0	1055.0	69.0	286
Minimum	0.0	340.4	0.0	0
Median	4.0	510.8	0.0	1.0
Mean value	3.6	573.1	3.4	29.9
Variance	3.6	26,979.7	132.9	4667.6
Standard deviation	1.9	155.8	11.6	69.0

There are 12 entities with keyword mentions above the mean value by small hospitals (12%) and 10 by large hospitals (19.6%). 85 small hospitals (85%) do not have any knowledge-related keyword mention on their websites. With large hospitals, this relates to 22 (43.1%).

As indicated, the homogeneity of data related to size is quite high by small hospitals and lower by large hospitals. Similarly, the homogeneity of values for keyword mentions is lower by large hospitals (being quite low for small hospitals, too). This indicates that there are notable differences in how the hospitals approach their online communication, using knowledge terms.

Further analysis was used to find out whether the size, location, existence of the one-page website or portal-style website, or the existence of an own domain affect the use of knowledge related terms on the websites of the hospitals (dependent variables 5 to 10).

The results of the chi-square test of independence to analyse the two-way contingency table for each variable combination to determine whether there is an association between two variables of interest is shown in Table 2. Fisher’s Exact Test was used to validate the results of the Chi-Square test.

**Table 2:** Post-hoc analysis for the significant results

Variables Tested	Chi-Square, df	*p-*value	More than Expected	Fewer than Expected
Size vs MentionedK	26.67, 1	< .001*	Large, Yes	Large, No Small, Yes
Size vs MentionedKC	23.86, 1	< .001*	Large, Yes	Small, Yes
Size vs MentionedKR	18.29, 1	< .001*	Large, Yes	Small, Yes
Size vs MentionedKS	15.61, 1	< .001*	Large, Yes	Small, Yes
Size vs MentionedKM	8.51, 1	.004*	Large, Yes	N/A
Size vs MentionedTotal	24.51, 1	< .001*	Large, Yes	Small, Yes
State vs MentionedK	24.87, 7	< .001*	Vic, Yes	WA, Yes
State vs MentionedKC	16.07, 7	.02**	Vic, Yes	N/A
State vs MentionedTotal	19.88, 7	.006*	Vic, Yes	N/A
Dom vs MentionedK	59.33, 3	< .001*	3^rd^, Yes 2^nd^, Yes	2^nd^, No
Dom vs MentionedKC	35.09, 3	< .001*	3^rd^, Yes 2^nd^, Yes	N/A
Dom vs MentionedKR	22.60, 3	< .001*	3^rd^, Yes 2^nd^, Yes	N/A
Dom vs MentionedKS	44.03, 3	< .001*	2^nd^, Yes	2^nd^, No
Dom vs MentionedKM	17.17	.001*	2^nd^, Yes	N/A
Dom vs MentionedTotal	49.45, 3	< .001*	3^rd^, Yes 2^nd^, Yes	Yes, No
P or O vs MentionedK	89.76, 2	< .001*	O, No P, Yes	O, Yes P, No
P or O vs MentionedKC	48.98, 2	< .001*	P, Yes	P, No
P or O vs MentionedKR	25.97, 2	< .001*	P, Yes	N/A
P or O vs MentionedKS	44.50, 2	< .001*	P, Yes	P, No
P or O vs MentionedKM	13.17, 2	.001*	P, Yes	N/A
P or O vs MentionedTotal	68.42, 2	< .001*	O, No P, Yes	P, No

A p-value with an * indicates significance at the 0.01 level while ** indicates significance at the 0.05 level

There were three variable combinations yielding non-significant results indicating that there is no association in State versus MentionedKR (χ^2^(7) = 6.71, *p* = 0.46); State versus MentionedKS (χ^2^(7) = 13.45, *p* = 0.06); and State versus MentionedKM (χ^2^(7) = 7.56, *p* = 0.37). For the significant results, indicating an association between the categories of the two variables, a post-hoc analysis was applied to identify the categories producing the significant result, i.e. which category combination(s) were significant in each case (Table 2).

## Discussion

Australian public hospitals include knowledge terminology on their websites (Research Question 1). There are significant differences between the level of online communications between Australian public hospitals, whether between individual entities within one group by size (large, small) or between the two different size groups (Research Question 2). Large hospitals are generally more active in this area, however, there are still many large hospitals who do not include many references to knowledge terms on their websites. Small hospitals, on the other hand, usually do not operate a website on their own domain and their websites are just a one-page presentation in many cases. Some of the small hospitals can lead by example and they represent an inspiration for many large hospitals who do not operate their website using their own domain name, and/or do not refer to knowledge-related terms at all.

Other factors significantly affect the level of knowledge-related online communications (Research Question 3). These include: 1) The location – significantly fewer hospitals based in Victoria do not mention the term ‘knowledge’ (single keyword), knowledge creation, and knowledge in general (total of documents containing any of the keyword combinations) at all; 2) Own domain name – hospitals using their 2^nd^ or 3^rd^ level domains communicate about knowledge significantly more compared to those being a part of the network website portal); 3) Having a larger portal-style website rather than a one-page presentation-style website means that the number of websites containing the examined keywords is larger.

Previous research conducted by [29] revealed that Australian companies consider it important to communicate their values regarding the importance of knowledge generation, sharing, and management in their publicly available websites. More than half of the companies (54.7%) had at least one document that contains the knowledge-related term(s). The current research has shown that this percentage is lower for Australian public hospitals (29.1%). The percentage of entities not having any relevant keyword mentions was 24.0% by the large Australian companies and 43.1% by large Australian public hospitals; 88.0% by Australian SMEs and 85.0% by small Australian public hospitals. Thus, the situation in smaller organisations is similar in this cross-sector comparison. However, large Australian companies use more knowledge terms on their websites when compared to large Australian public hospitals (Research Question 4).

In this research, the strong correlation was found between the size of a public hospital and the communications of knowledge-related terminologies with their online audience. The *p-*value indicating the significance at the 0.01 level means that the large hospitals use these terms significantly more than small hospitals. Of the six analysed relationships, five have a *p-*value < 0.001. This correlates with the expectations defined. Large organisations are naturally developing into the state where they require optimised processed related to cooperation between people located in various departments and across their managerial structures. It can be expected that larger hospitals would have certain knowledge management processes in place that are of higher maturity levels than those of small hospitals. In small hospitals with few staff members, the exchange of information can be more verbal, less structured and formalised. Large hospitals would also operate with more resources, both in the management and marketing operations area. They also have a greater variety of stakeholders, patients and other potential recipients of their communications, and therefore they need to be more professional and complex. Some of the hospitals stand out in their level of knowledge-related online communications. It would be interesting to determine the reasons for this performance and examine the alignment with their commitment to managing their knowledge.

With regards to the difference between states, an interesting finding shows that Victorian hospitals include more knowledge terminology on their websites more than hospitals from other states. The relationship between the variable ‘State’ with values ‘VIC’ and the number of hospitals mentioning the keyword ‘knowledge’ at least once, as well as the number of hospitals with at least one mention of any of the keywords in the four examined knowledge areas, shows the significance at the 0.01 level.

Some of the hospitals were using the domain provided by the network, whereas the others operated on their own domain, whether it was the completely independent 2^nd^ level domain or the 3^rd^ level domain. The results show that the latter is the case and that hospitals having a website on their own domain do include knowledge-related terms on their websites more than those being part of a network website portal. For some of the knowledge areas, this strong relationship between having an own domain and the online communications about knowledge was only related to having an own 2^nd^ level domain (knowledge sharing and knowledge management areas). For the other four variables, the significance was very strong even for the 3^rd^ level domain.

Finally, it was confirmed, that significantly more hospitals that operate a portal style website mention the knowledge-related terms at least once, than those with only a one-page website. This can be expected, despite the fact that a one-page website could also contain a number of linked PDF documents that might include this terminology.

## Conclusions

The research revealed a significant correlation between the size of the hospital and the websites containing knowledge-related keywords. The relationship between the existence of a knowledge culture and the quality of services has been documented in the literature. The current study provides insights into the communication of such a knowledge focus on the websites of Australian public hospitals. The results reveal the differences between the hospitals of various sizes, types and locations and represent empirical evidence indicating the existence of a knowledge focus that might impact the performance of Australian public hospitals.

### Limitations of the study

Only public hospitals were included in this study. A limited number of knowledge terms were selected for inclusion in this study. No distinction was made between the specific types of knowledge used in public hospitals, such as clinical and administrative knowledge. Only documents that are published on the websites of the hospitals were included in the study.

### Further research

Opportunities exist for future research, namely to: 1) Enhance the methodology by adding new general keywords such as collaborate, collaboration, cooperate, cooperation, expertise, share ideas, share findings, research etc.; 2) Add specific knowledge types as keywords, e.g. medical knowledge, clinical knowledge, patient knowledge, theoretical knowledge, nursing knowledge, specialist knowledge, drug knowledge, scientific knowledge etc.; 3) Focus on longer publications (e.g. reports, research results) that are often common for the local hospital network and include them in the research, despite the fact that they are not published on the website of a particular hospital; 4) Include communications on social media and other channels in the research; 5) Include private hospitals in the research sample to gain insight into their websites and compare the differences and similarities with public hospitals.

## Data Availability

The datasets used and/or analysed during the current study are available from the corresponding author on reasonable request. Not applicable

## Abbreviations

IT: Information technologies
GDP: Gross domestic product
KM: Knowledge management
KI: Knowledge-intensive
ACT: Australian Capital Territory
NSW: New South Wales
NT: Northern Territory
QLD: Queensland
SA: South Australia
TAS: Tasmania
VIC: Victoria
WA: Western Australia
P: Value indicates that the website is a complex portal consisting of multiple pages
O: Value represents a one-page website which does not have a deeper structure
Dom: The existence of an own domain was identified
K: Total number of documents containing the keyword ‘knowledge’
KC: Total number of documents with keywords related to knowledge creation
KR: Total number of documents with keywords related to knowledge retention
KS: Total number of documents with keywords related to knowledge sharing
KM: Total number of documents with keywords related to knowledge management
MentionedK: Binary variable—the phrase K was mentioned (1) or not (0)
MentionedKC: Binary variable for the count of KC
MentionedKR: Binary variable for the count of KR
MentionedKS: Binary variable for the count of KS
MentionedKM: Binary variable for the count of KM
